# Supplementary material for: Transcriptome Analysis to Identify Responsive Genes under Sublethal Concentration of Bifenazate in the Diamondback Moth, Plutella xylostella (Linnaeus, 1758) (Lepidoptera: Plutellidae)
Source: Int J Mol Sci. 2022 Oct 29;23(21):13173. doi: 10.3390/ijms232113173 (PMC9656211; doi:10.3390/ijms232113173)
Supplement: Supplementary file 1 [file ijms-23-13173-s001.zip › Figure S1.pdf]

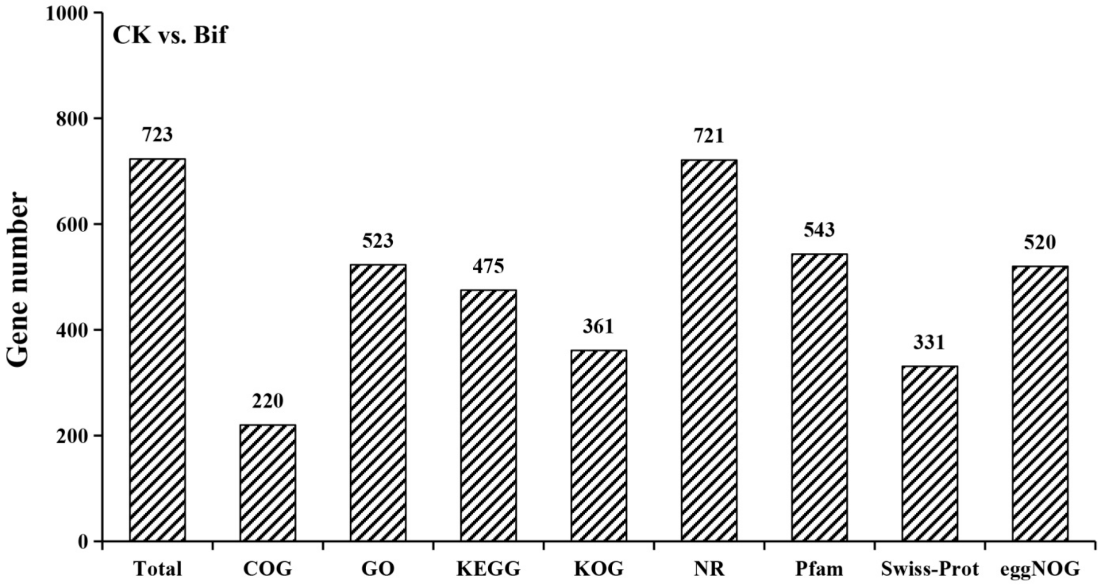


**Figure S1.** The annotated of differentially expressed genes (DEGs) based on different databases, including COG (Clusters of Orthologous Groups of proteins), GO (Gene Ontology), KEGG (Kyoto Encyclopedia of Genes and Genomes), KOG (EuKaryotic Orthologous Groups), NR (NCBI nonredundant protein sequences), Pfam (Protein family), Swiss-Prot (A manually annotated and reviewed protein sequence database) and eggNOG databases.
